# The Directiveness that Dare Not Speak Its Name. Views and Attitudes of Polish Clinical Geneticists toward the Nondirectiveness Principle

**DOI:** 10.1007/s11673-022-10202-x

**Published:** 2022-09-01

**Authors:** Weronika Chańska, Katarzyna Grunt-Mejer

**Affiliations:** 1 Department of Philosophy and Bioethics, Jagiellonian University Medical College, Michałowskiego 12, 31-126 Kraków, Poland; 2 Faculty of Psychology and Law in Poznań, SWPS University of Social Sciences and Humanities, Kutrzeby 10, 61-719 Poznań, Poland

**Keywords:** Genetic Counselors, Prenatal Diagnosis, Attitudes, Beliefs, Nondirectiveness, Clinical Ethics

## Abstract

Nondirectiveness is widely regarded as an important principle of genetic counseling. However, numerous studies have indicated that the use of this principle and its content itself are subject to controversies. The present study aimed to verify how the nondirectiveness principle is defined by Polish geneticists, the extent to which it is considered the main principle in clinical practice, and the situations in which geneticists see the positive value of the directive action. Using quantitative and qualitative methods, the study compared the abstract declarations of the directiveness validity and the scope of this principle with the declaration of action in situations close to reality (case scenarios). The results showed that the high rank assigned to the nondirectiveness principle does not translate into the conviction about the absolute obligation to use it in clinical practice. Polish geneticists are inclined to restrict the scope of patients’ choices when these are outside of their definition of medical standard. Strong medical paternalism manifests itself particularly in invasive prenatal diagnostics, where geneticists play the role of gatekeepers. In this study, we offer hypotheses about the sources of these attitudes by analyzing the current cultural and legal context of Poland.

## Introduction

Nondirectiveness is widely regarded as the main principle of genetic counselling (Wertz and Fletcher 1988). Nondirective counselling is currently assumed to have two major elements. The first is the provision of accurate, full, and unbiased information that may be used by individuals and their families for decision-making. The second is an understanding, empathic relationship that offers guidance and assistance for people to work toward their own decisions (World Health Organization Human Genetics Programme 1998).

Although the principle of nondirectiveness plays a central role in genetics and is broadly endorsed by those providing genetic counselling, it has been criticized from a number of perspectives, which question the following: the practical feasibility of making genetic counsel completely nondirective (Bosk 1993; LeRoy 1993; Marks 1993; Sorenson, Kavanagh, and Mucatel 1981), the scope of duties of the persons who provide genetic advice and their role toward the patient (Kessler 1997; Weil 2003; White 1997), and the validity of morally neutral attitudes held by geneticists in issues concerning the welfare of other individuals and society (Caplan 1993; Yarborough, Scott, and Dixon 1989). However, despite these controversies, there is a strong conviction that the behaviour of a genetic counsellor should support patients in making their own decisions. This principle is particularly well established in the case of reproductive decisions (World Health Organization Human Genetics Programme 1998).

Empirical studies have shown that genetic counsellors acknowledge the nondirectiveness principle in counselling (Bartels et al. 1997; Wertz and Fletcher 1988). At the same time, they do not always use this principle, and sometimes are even aware of acting in a directive way (Bartels et al. 1997). This may mean that although nondirectiveness is the main objective of genetic consultations, other significant values may affect a counsellor’s decision to act in a directive way.

Several works indicate that individual factors and social conditions may also determine the attachment to the nondirectiveness concept and its application in practice. One of these factors is gender; studies have shown that women declared stronger attachment to nondirectiveness and lower directiveness in practice (Curlin et al. 2007; Wertz 1994; Wertz and Fletcher 1988). A higher level of nondirectiveness is further correlated with lower professional experience, lower religiousness (Curlin et al. 2007), and liberal political opinions (Wertz and Fletcher 1988). Cohen et al. (1997) state that personal convictions may substantially affect the method by which genetic advice is provided and may interfere with nondirectiveness, particularly where this principle is of importance–according to its own theory. Certain studies indicate that education plays an important role in the acceptance of the patient’s autonomy—counsellors with an educational background other than medical are more sensitive to the policies of autonomy and privacy than physicians (Pencarinha et al. 1992)

The level of directiveness may also be affected by historical and social factors. Despite the common declaration of support for the idea of nondirectiveness, counsellors hailing from the countries of the former socialist block have been shown to display a stronger tendency to select directive action in clinical practice compared to those from other countries (Wertz and Fletcher 1988). A similar relationship was recorded when counsellors from East and West Germany were compared, of which the counsellors from the eastern states exhibited a higher level of directiveness and increased acceptance for pregnancy termination (Cohen et al. 1997). Marteau et al. (1994) observed some differences between the geneticists from Great Britain, Germany, and Portugal, and therefore concluded that nationality may be relevant for both the force of directiveness and its direction. The authors reported that German counsellors leaned toward advising against abortion, while Portuguese counsellors proposed this intervention. In this background, it seems interesting to analyse how nondirectiveness is defined and viewed in the field of genetic counselling and identify the extent to which this principle is respected in the counselling process in Poland, considering the current cultural characteristics of the country.

### Prenatal Genetic Counselling Standards in Poland

In Poland, qualified physicians specialized in clinical genetics are eligible to provide genetic counselling. Similar to general medical education, the majority of geneticists are women. Clinical genetics is a relatively new specialty. It was introduced in 1995 and modelled on specialization training in other medical fields. Access is restricted to physicians and the training programme is dominated by obligatory theory classes. These classes are supplemented with a clinical internship, which is organized independently by each person taking the training, selecting from a list of certified institutions. As a consequence, training in the field of clinical genetics in Poland is mostly theoretical and aims at improving medical knowledge. However, practical skills associated with the psychosocial aspects of genetic counselling are not focused on in the training programme, and their acquisition mostly depends on the resourcefulness of the person getting trained in the specialty. Therefore, the lack of standardization in this scope disposes a future clinical geneticist to typically assume the work mode established in the institution where he/she undertakes the internship.

Handbooks used in clinical genetics training (both Polish and translations of foreign publications) usually do not contain large amounts of information on the practical aspects of genetic counselling. They also devote only a little space to the objectives and normative principles that form the basis of counselling. Furthermore, the content presented therein is typically limited to general normative recommendations (such as nondirectiveness and respect for the patient’s autonomy). However, these books do not offer specific advice on how to realize the principles assumed in clinical practice. Polish clinical geneticists primarily confine themselves to declaring general support for the ethical principles that are to be observed by a counsellor providing genetic advice. In contrast to geneticists in other countries, Polish genetic counsellors have not conducted any serious internal debate on the role and importance of nondirectiveness in counselling practice. The nondirectiveness principle is commonly asserted in Poland, but thus far no attempts have been made to define or translate it into more precise recommendations for clinical practice.

In Poland, prenatal diagnostics is performed as part of the general medical services. Access to a free prenatal examination is granted to women who are at an elevated risk of giving birth to a child with a genetic condition. This group is characterized by 1) a maternal age of thirty-five years or older on the estimated date of delivery, 2) a positive aneuploidy screening result (risk cutoff level of 1:300), 3) a known family history of genetic disorders, and 4) presence of anomalies on ultrasound. The recommended workup assumes performing noninvasive screening tests, followed by invasive prenatal diagnosis. Obtaining a referral is the prerequisite for performing the invasive testing, which must be preceded by a consultation with a geneticist.^1^

### Importance of Nondirectiveness Where Genetic Counsellors Act as Abortion Gatekeepers

Poland has the strictest abortion law among European countries. When our study was conducted, the law allowed the termination of pregnancy only in the following three cases: 1) when the pregnancy poses hazards to the life or health of the mother, 2) when the pregnancy is the result of criminal act, and 3) when severe fetal malformations are predicted. In the latter case, the law stated that pregnancy can be terminated “where prenatal tests or other medical findings indicate a high risk that the fetus will be severely and irreversibly damaged or suffering from an incurable, life-threatening disease.”^2^ The basis for applying for pregnancy termination was obtaining a medical certificate confirming that the fetus will have malformations. This document should be issued either by a physician specializing in genetics if the malformation is determined by genetic testing or by a physician specializing in obstetrics and gynaecology if the malformation is determined based on a fetal ultrasonography examination. Issuing the certificate does not require obtaining certainty of the fetal malformation; it is sufficient to determine its high likelihood. Furthermore, the law does not accurately define the terms “severe, irreversible damage” or “incurable, life-threatening disease,” and their meaning should be determined by clinical practice. In Poland, this practice imposes pregnant women with further-reaching requirements than those provided for in the act. In particular, it is assumed by many Polish physicians that the assessing terms used by the legislature, such as “severe” and “life-threatening,” are subject to personal assessment by the physicians and the importance of these terms depends on their personal opinion about the given malformations. It is also relatively common to assume that the opinion of the pregnant woman can be neglected in this regard. Thus, obtaining the certificate of pregnancy termination is a matter of discretion to a significant extent, depending on the goodwill and the personal beliefs of the physician (Zaręba et al. 2017). Against this background, special importance is gained by the validity of the nondirectiveness principle in the case of the given physician, and the awareness of one’s own attitudes and their possible impact on not only the patient’s approach to the perspective of continuing or terminating the pregnancy but also the sole possibility of making this decision.

International comparative research including Poland by Wertz and Fletcher (2004) was carried out between 1993 and1995, that is, the period before the clinical genetics specialty was introduced in the country. Of the surveyed participants, 52 per cent were physicians, while midwives formed the second largest group, accounting for 30 per cent of the sample. In this study, the majority of the surveyed individuals were women (71 per cent). Thus, people with different educational backgrounds and varying levels of experience were surveyed rather than the current population of clinical geneticists. In addition, the study of Wertz and Fletcher was carried out when a different scenario of rights prevailed in Poland (until 1993, abortion was allowed in the country if demanded by the difficult life situation of the woman, which contributed to its actual wide accessibility). Therefore, it is necessary to take another look at the views and values held by the persons who currently provide genetic counselling, including consultations preceding the invasive prenatal testing.

### Study objectives

In the light of the absence of strictly determined professional standards specifying how prenatal genetic counselling is conducted and the reports on the high level of discretion in terms of respecting the reproductive choices of pregnant women, we have formulated the following research questions:

1. Is the nondirectiveness principle seen as significant among genetic counsellors?
2. How do genetic counsellors understand the principle?
3. Do genetic counsellors accept situations when being directive is a justified action?
4. What are the convictions of Polish geneticists on the acceptability of performing invasive prenatal testing? Do geneticists allow invasive testing in the situations when its result does not have any significance for the pregnancy outcome?
5. Whose opinion, according to genetic counsellors, should be taken into account in the process of deciding on the acceptability of pregnancy termination?
6. What are the convictions of Polish geneticists on the acceptability of pregnancy termination in the event of specified fetal malformations?

## Methods

### Participants and Procedure

This study was approved by the Bioethics Committee of the Jagiellonian University. Printed questionnaires were handed out during the 5th Polish Genetics Congress in Łódź (held between September 19 and 22, 2016), while the electronic version (available on the ankietka.pl website) was sent to genetic clinics listed by the Polish Society of Human Genetics and to the voivodeship clinical genetics counsellors who forwarded it to the genetic institutions operating in their area. All persons who expressed interest to participate in the survey provided informed consent before completing the questionnaire.

A total of twenty-one filled print forms and fourteen electronic surveys were obtained. All thirty-five respondents were clinical genetic specialists, which accounted for 31 per cent of all geneticists in Poland. A considerable majority of the surveyed participants responded to all the questions, and only in very few cases did the participants not provide any response to a question. In such a situation, we provide the number of people who answered such a question.

According to the data provided by the national clinical genetics counsellor, 114 clinical genetics specialists were registered in 2016. A significant majority of them (68 per cent, seventy-seven individuals) were women, while men comprised 32 per cent (thirty-seven people). The survey results approximately reflected this proportion, with a minor overrepresentation of women. The sample demographics are reported in Table 1.

**Table 1:** Sample demographics

Variable	N	%
Sex		
Women	27	77.1
Men	8	22.9
Work time as a clinical geneticist		
<5 years	5	14.3
5–10 years	9	25.7
11–15 years	10	28.6
16–20 years	4	11.4
21–25 years	2	5.7
>25 years	5	14.3
Additional medical specializations^a^		
Clinical genetics specified as the only specialization	19	54.3
Pediatrics	9	25.7
Laboratory genetics	2	5.7
Neonatology	2	5.7
Other:	4	11.4
Gynaecology and obstetrics	1	
Neurology	1	
Ophthalmology	1	
Internal medicine	1	
Other specialization without a name specified	3	8.6
Place of work (in the event of employment in several workplaces, the surveyed were asked to provide the nature of the main place of work)		
(1) Nonprofit-only entity (financed from public funds)	19	54.3
(2) Commercial-only entity	3	8.6
(3) Both commercial and nonprofit entity	13	37.1

^a^Some of the surveyed participants specified more than one additional specialization, meaning that the total percentage is over one hundred.

### Instrumentation

The first part of the study questionnaire had questions on demographics. In the second part, the respondents were asked to indicate the extent to which they considered nondirectiveness as important in their clinical practice, how they define directiveness, and under what conditions they believe being directive is justified. They were also asked whether a given reason is sufficient to refer for invasive diagnostics or whether the given fetal condition (such as Down syndrome, cystic fibrosis, achondroplasia, Klinefelter’s syndrome, Duchenne muscular dystrophy, or Edwards’ syndrome) can be assumed as sufficiently severe to issue a referral for pregnancy termination. For clarification, the detailed questions are presented together with the results of the corresponding analysis.

Our questionnaire combined closed-ended as well as open-ended questions. Some of these questions required selecting one of the answers provided by us, while the open-ended ones required the participants to provide their own answer. Apart from the questions in which the surveyed were asked to declare whether they act according to the specific principle named by the investigators, the questionnaire provided clinical examples (case scenarios) involving the same principles, yet in a less abstract context. For these questions, the participants were asked to describe how they would act in such situations (self-report on their behaviour in counselling scenarios). This enabled a comparison of the proceeding methods declared by the respondents to their actual attitude with regard to the specific clinical examples.

### Data Analysis

The IBM SPSS Statistics software was used to perform quantitative analyses. The answers were analysed in terms of sex, professional experience (lower (up to fifteen years) and higher (fifteen years and above)), specialization (pediatrics course and others), and working only in a national vs. (also) a private institution. Due to the nominal nature of all variables, the ϕ (Phi) test was applied for the analysis.

Qualitative data obtained during the study were examined using thematic analysis (Braun and Clarke 2006). Transcripts were reviewed by the first author (WC) and manually coded for broad themes. A codebook was created using the codes emerging from the transcripts. A second coder (KGM) reviewed a subset of transcripts using the codebook and added any newly identified codes. Minor discrepancies in coding were discussed by the two coders and resolved. Atlas.ti software was used to analyse the qualitative data.

## Results

### Nondirectiveness as a Significant Character of Genetic Counselling

The surveyed participants were asked to indicate whether they considered nondirectiveness as an important character of genetic counselling. They selected an answer from a five-point Likert scale (1 = very important, 5 = not important). A significant majority (71.4 per cent, n = 25) indicated that, according to them, nondirectiveness is a very important character of genetic counselling, while the remainder (25.7 per cent, n = 9) considered it as important. None of the surveyed individuals selected other options (such as less important or unimportant). Sex, professional experience, specialization, and place of work were not found to influence the respondents’ answers in a statistically significant manner.

### Understanding Nondirectiveness

Two questions were related to the content of nondirectiveness. The first was an open-ended question which asked the surveyed participants to formulate their own definition of the nondirectiveness principle. Definitions were provided by thirty-one people, which were subject to thematic analysis, after assigning them with suitable codes. On this basis, five main components of the principle were distinguished. The answers of the respondents typically contained more than one of the distinguished categories. The question and the example answers given by the participants for each category are provided in Table 2.

**Table 2:** “Describe as accurately as possible what does nondirectiveness mean with regard to genetic counseling to you?”—answer analysis

Category	Example answers
Providing patient^a^ with full and true information on her situation (n = 21)	“Presenting the patient with all possible diagnostic/therapeutic measures” “Presenting information as objectively as possible and in line with the current state of knowledge”
Lack of genetic counsellor impact on patient’s decisions (n = 21)	“Not imposing personal opinion, not ordering a specific action” “Not enforcing any action by the patient, not imposing own opinions”
Enabling patient to make an independent decision (n = 17)	“Patient has the right to make her own independent decision” “The decision is up to the patient regardless of the specialist’s views”
Restraining from expressing one’s own opinion, how they would act if they were in patient’s position (n = 10)	“Not answering the question ‘what would you do if you were in my position?’”; “No impact of one’s own beliefs and worldview on the manner of conducting genetic counseling”
Supporting patient in making her own decisions (n = 2)	“Support provided by physician, independently of his personal opinion” “Providing patient with support in the realization of the decision made”

^a^Polish specialists use the term “patient” for a person who receives genetic advice. The word “client” has not been adopted in Poland because most geneticists in the country believe that it signifies an incorrect association with the service providing. According to them, genetic advice is a medical advice that is based on fiduciary relationship. Considering this linguistic tradition, we use the term “patient” consistently throughout this article to signify a person receiving genetic advice.

The second, closed-ended question asked the participants to more accurately determine the types of behaviour that remain within the scope of directiveness. The respondents were presented with six example behaviours of a genetic counsellor, in which certain information was concealed from the patient or the patient was told how she should act. From these, the surveyed should indicate the examples that they felt as directive.

None of the examples provided in the questionnaire were identified as directive by all surveyed individuals. The least uncertainty in terms of directive nature was raised by the statement that the patient should not terminate the pregnancy, whereas providing the patient with either overly optimistic or pessimistic diagnostic information was the least frequently selected as directive (selected by 65.7 per cent of the respondents (n = 23)). The question and the percentage of the answers given by the participants are presented in Table 3.

**Table 3:** “Of the below descriptions, mark those which you find to be examples of directiveness when providing genetic advice”—answer presentation

Genetic counsellor behaviour	Indicated as directive behaviour	
	%	Number
Telling the patient that she should not terminate the pregnancy	85.7	30
Responding to patient’s question: “What would you do if you were in my position, doctor?”	77.1	27
Telling the patient to take genetic test	74.3	26
Nondisclosure of some information to the patient	71.4	25
Providing the patient with overly optimistic diagnostic information	65.7	23
Providing the patient with overly pessimistic diagnostic information	65.7	23

A significant result was observed for sex in the case of selecting the answer as directive to the patient’s question: “What would you do if you were in my position, doctor?” (phi =.35, *p* = .037, n = 35). Half of the surveyed men declared that they did not see this behaviour as directive, whereas only 14.8 per cent of women gave this answer. No statistically significant differences were found for other dimensions of directiveness or when another independent variable was included in the analysis.

### Acceptability of Directiveness

The surveyed were asked whether they could think of any situations when directiveness from the genetic counsellor is advisable. The participants who provided an affirmative answer were asked to describe such situations. Of all the respondents, 63 per cent (n = 22) answered that there are certain situations when the geneticist can exhibit a directive behaviour. Only 37 per cent (n = 13) believed that the nondirectiveness principle has an absolute character. No differences were found with respect to sex or additional specialty for this answer. However, significant differences were observed between persons with lower and higher experience (phi = .035, *p* = .039, n = 35), and those with a higher professional experience more frequently indicated that nondirectiveness has an absolute character. A significant result was obtained for persons working in commercial and nonprofit organizations—respondents employed in commercial organizations more frequently indicated that directiveness is acceptable in certain situations (phi = .035, *p* = .039, n = 35).

The responses of people accepting the directive behaviour of a genetic counsellor were assigned appropriate codes and subject to thematic analysis. Four situation groups in which the surveyed accepted directiveness exhibited by a geneticist were distinguished as follows: 1) situations in which leaving the decision to the patient may lead to negative outcomes, 2) situations in which the patient makes irresponsible procreation choices, 3) situations in which the patient is incapable of making an independent decision, 4) situations in which the patient’s choice violates the professional standard. The question and the contents of each category are presented in Table 4.

**Table 4:** “In your opinion, are there situations in which directiveness on the part of a genetic counselor is advisable? If yes, provide what kind of situations are those”—response analysis

Category	Situation description
Situations in which leaving decision to patient may have negative outcomes (n = 14)	Patient’s decision may have negative outcomes for her life or health (n = 7) Directiveness of geneticist helps improving patient’s health status—implementation of prophylaxis or undertaking early testing (n = 4) Actions of patient violate the good of her relatives (n = 3)
Patient makes irresponsible procreation decisions (n = 8)	Pregnancy poses hazard to the life or health of the mother (n = 4) Patient makes irrational procreation decisions (n = 2) It would be advisable to perform prenatal diagnostics for the good of the fetus (n = 1) When a woman wants to terminate pregnancy due to a mild fetal defect (n = 1)
Patient is incapable of making independent decision (n=5)	Incapability to make decision due to limited cognitive capabilities of patient (n = 3) Patient behaves irrationally (n = 1) Telling patient what she should do enables her to take appropriate distance to her own situation (n = 1)
Decision to be made by the patient violates professional standard (n = 2)	Patient’s choices would be against the law (n = 2) Actions violating professional standards (n = 1)

### Sufficient Reasons to Direct for Invasive Diagnostics

The participants were presented with a list of six situations and asked to mark all those which they thought are sufficient reasons to refer a pregnant woman for an invasive prenatal examination. The situations were as follows: 1) the pregnant woman was aged over thirty-five, 2) the pregnant woman had positive aneuploidy screening results or anomalies identified on ultrasound, 3) the pregnant woman had a known family history of genetic disorders, 4) the pregnant woman had extreme anxiety, and 5) the pregnant woman had no medical indications but nonetheless was willing to undergo an invasive prenatal examination. Table 5 presents the answers provided by the respondents.

**Table 5:** Sufficient reasons to issue a referral for a pregnant woman for an invasive prenatal examination

	%	Number
Positive aneuploidy screening results or anomalies identified on ultrasound	97.1	34
Known family history of genetic disorders	91.4	32
Extreme anxiety of a pregnant woman	48.6	17
Pregnant woman aged above thirty-five	28.6	10
At the request of the pregnant woman	22.9	8

Sex, level of professional experience, type of specialty, and nature of the institution were not found to differentiate the responses given to the above question.

### Acceptability of Invasive Testing When its Result Does not Have any Significance for Pregnancy Outcome

The next question was constructed to verify whether the consultants regulate the access to invasive examinations due to their convictions about the aim of such examinations. This manifestation of possible paternalism by the respondents was measured by asking whether an invasive prenatal examination should be performed for women who categorically oppose the pregnancy termination. The question indirectly concerned the participants’ views on the objective of performing prenatal testing and the circumstances justifying the associated risk to the life of the fetus. It was assumed that persons aiming at maximizing the good of the fetus (even at the cost of the mother’s right to information) would be against performing prenatal examinations in those individuals who oppose pregnancy termination regardless of the examination result. Of thirty-five respondents who answered this question, 25.7 per cent (n = 9) indicated that such examinations should not be conducted in those women. This suggests that some geneticists view prenatal testing primarily in the context of the possible decision on pregnancy termination. At the same time, they believe that the associated risk of pregnancy loss is severe enough that such examinations should not just be performed for understanding the status of the fetus. This response was influenced by sex at the level of statistical tendency—nine women disapproved of examinations in this situation (phi = .32, *p* = .058, n = 35). The remaining controlled characteristics did not differentiate the response to this question.

Our team wanted to verify if the respondents’ declarations aligned with their actions in a specific situation close to reality. The latter part of the questionnaire included the case description of a patient aged forty-two, whose first child was born with Down syndrome. The woman rules out pregnancy termination, but she wants to know if her second child will also suffer from the disease. At the same time, she declares that she would not terminate the pregnancy irrespective of the examination outcome. The surveyed participants were asked to describe in their own words what they would do in the described situation. Only one person (2.9 per cent) declared that she would deny the referral.

The result was compared with the earlier theoretical declarations. This analysis included only those questionnaires that had answers to both questions (n = 34). All the individuals who did not oppose performing invasive testing in women who will continue pregnancy regardless of investigation results declared that they would issue a referral for the examination in this concrete situation. Of the eight people who declared that this examination should not be performed in women not willing to terminate the pregnancy, 88 per cent (n = 7) changed their minds in response to a real-life scenario. Only one person remained true to the original declaration on the inappropriateness of performing prenatal testing under such circumstances. This revealed that an abstract declaration does not always translate into action in a specific case.

### Who Should Decide on the Acceptability of Pregnancy Termination?

The surveyed were asked if they provide consultations on prenatal testing in their practice. A majority of respondents (82.9 per cent, n = 29) provided an affirmative answer to this question. In an attempt to examine if the geneticists’ views on the acceptability of pregnancy termination had any impact on the procreation choices made by patients, the respondents were asked to specify who should assess whether the fetal malformations are severe enough to enable legal termination of pregnancy and were provided six options. Table 6 presents the answers of the respondents to this question.

**Table 6:** “In your opinion, who should determine whether the malformation identified in fetus is severe enough to enable termination of pregnancy?”—answer analysis

Who should decide	Percentage and number
1. Special committee comprising (percentage of indications among the individuals who marked the answer “special committee”):	54.3 (n = 19)
• Geneticist	100 (n = 19)
• Gynaecologist or obstetrician	94.7 (n = 18)
• Neonatologist	47.4 (n = 9)
• Pediatrician	36.8 (n = 7)
• Other specialists in the field of medicine (surgeon, cardiologist, nephrologist, specialist in the field of metabolic diseases, and neurologist)	31.6 (n = 6)
Psychologist	21.1 (n = 4)
Ethicist	15.8 (n = 3)
Roman Catholic priest	5.3 (n = 1)
2. List of malformations and diseases	28.6 (n = 10)
3. Gynaecologist opinion is sufficient	2.9 (n = 1)
4. Geneticist opinion is sufficient	2.9 (n = 1)
5. Opinion of the pregnant woman is sufficient	2.9 (n = 1)
6. Other individuals (percentage of indications among the individuals who marked the answer “other individuals”)	8.6 (n = 3)
Geneticist or gynaecologist	66.7 (n = 2)
Pregnant woman	33.3 (n = 1)
Psychologist	33.3 (n = 1)
Specialists in the field of the disease	33.3 (n = 1)

Viewing the Given Disease as a Sufficient Reason for Issuing Statement About the High Likelihood of Severe and Irreversible Fetus Impairment or an Incurable Disease Threatening the Life of the Fetus

To identify the severity level that the counsellors felt was sufficient to issue the statement on the high likelihood of severe and irreversible fetus impairment or an incurable disease threatening the fetal life (the Statement), six disorders of genetic origin with different levels of physical and mental disability were selected. The participants were asked the question: “In your opinion, is detecting [disease name] in fetus a sufficient reason for issuing the Statement?” In the field “disease name,” the disorders appeared in the following order: Down syndrome, cystic fibrosis, achondroplasia, Klinefelter’s syndrome, Edwards’ syndrome, and Duchenne muscular dystrophy. None of the stated diseases was selected by all of the surveyed geneticists as a sufficient reason for termination of pregnancy. Edwards’ syndrome was the most commonly selected as a sufficient reason for issuing the statement authorizing pregnancy termination, whereas Klinefelter’s syndrome was selected least frequently. The detailed results are presented in Table 7.

**Table 7:** Disorders assumed as severe enough to issue the Statement—in order from the most commonly selected

Condition	%	Number
Edwards’ syndrome	88.6	31
Duchenne muscular dystrophy	68.6	24
Down syndrome	65.7	23
Cystic fibrosis	37.1	13
Achondroplasia	25.7	9
Klinefelter’s syndrome	2.9	1

It was also analysed if the idea that a given disease is severe enough to issue the statement was influenced by sex, professional experience, specialization, and the nature of the institution. Statistically significant results were obtained for cystic fibrosis (phi = .43, *p* = .012, n = 35) and Down syndrome (phi = .43, *p* = .012, n = 35) with respect to professional experience. Persons with a shorter experience more frequently indicated that the listed diseases are sufficient for issuing the statement.

In the case of sex, a relationship at the level of tendency was observed for achondroplasia (phi = .32, *p* = .058, n = 35). None of the surveyed men stated that this disease is profound enough to justify issuing the statement.

In the case of specialty, a statistically significant result was obtained for Down syndrome (phi = .40, *p* = .018, n = 35). Respondents with pediatrics specialization significantly less frequently stated that this disease is sufficiently severe to issue the statement. At the same time, among those who had more than one medical specialty, only pediatricians and neonatologists indicated Down syndrome is not a sufficiently severe disorder to issue the statement.

The surveyed participants who were working solely at an institution financed from public funds more frequently indicated that cystic fibrosis meets the criteria for issuing the statement (phi = .43, *p* = .012, n = 35). Such a relationship at the level of tendency was also observed for Down syndrome (phi = .30, *p* = .072, n = 35).

## Discussion

All the surveyed geneticists declared that nondirectiveness is a very important or important feature of genetic counselling. This result significantly differs from the observations of Wertz and Fletcher (2004), who ascertained from their study that “the ‘non-directive’ counselling found in English-speaking nations is an aberration from most approaches found elsewhere” (50).

In our study, the respondents not only declared strong attachment to nondirectiveness, but when asked to provide their own description of the principle, they defined it in a similar manner as the international literature (Table 2). The significant components of nondirectiveness mentioned by the surveyed Polish geneticists were providing complete and adequate information to the patient, restraining from expressing personal opinions and affecting the decision of the person receiving the advice, and enabling the advised person to make an independent decision and supporting her in its realization. However, although the declarations of the surveyed were very similar to the nondirectiveness definition proposed by the World Health Organization (WHO), they emphasized the information elements of the counselling process and the lack of interference on the part of the geneticists. Among the declarations of the surveyed physicians, the conviction on the need to provide the advised person with assistance in making the appropriate decision was found to be less common. Only two surveyed individuals indicated that it is the geneticist’s obligation to provide the patient with support. This suggests that Polish geneticists have assumed the counselling model referred to by Kessler (1997) as the teaching model.

However, the commonness of the conviction of the value of nondirectiveness and the similar manner of defining the principle to English-speaking countries turned out to be relative when the geneticists were confronted with situations resembling clinical practice (Table 3). When the surveyed were provided with counsellor behaviour examples (undoubtedly directive), they did not frequently see them as directive. None of the given examples (including telling the patient that she should not terminate the pregnancy) was selected as directive by all of the surveyed geneticists. At the same time, an unexpectedly high percentage of the surveyed declared that they did not view manipulation of the information provided to the patient as a directive consultant behaviour. Close to 35 per cent of geneticists assumed that conscious provision of overly optimistic or overly pessimistic information to the patient does not violate the nondirectiveness standard of counselling. These results may support earlier research indicating that the declaration of the high value of directiveness is not always accompanied by the actual action (Brunger and Lippman 1995; Rapp 1988).

Responses to further questions confirmed that the high rank assigned to the nondirectiveness principle does not translate into the conviction about the absolute obligation to use it in clinical practice. Only 37 per cent of the surveyed participants believed that the principle has an absolute character, while the remaining 63 per cent accepted directive counsellor behaviour and assumed that it was even appropriate in a range of situations. The descriptions of situations justifying directive counsellor behaviour provided by the surveyed revealed paternalism rooted in the geneticist circles, which was manifested by the conviction that the autonomous choices of the patient should be supported only as long as those are “good”—compliant with the “common sense” and not violating anyone’s interests.

The surveyed Polish geneticists were also inclined to restrict the scope of reproductive choices in situations where these were outside of their definition of medical standard. The results indicated that access to invasive prenatal testing is, to some extent, rationed by geneticists. Their decisions on issuing or rejection of a referral for invasive prenatal examination reflect the conviction of the need to ensure a balance between the risk of the examination itself and the likelihood of fetal malformation. Only about one quarter of the respondents assumed the right of a pregnant woman to information on the health status of the fetus and her right to take the risk to obtain the information. A markedly higher part of the study group (close to 50 per cent) accepted issuing a referral for invasive prenatal examination in the case when extreme anxiety of the patient was the sole indication. This probably indicates that in such circumstances the surveyed were leaning toward considering the negative impact of prolonged stress on the health of the pregnant woman. Another explanation can be the fact that this very circumstance from the beginning of invasive prenatal testing in Poland has been indicated by the pioneers of such examination (individuals who are influential in the geneticist circles) as the one justifying the test performance. Up until this day—although the premise is not listed among refund indications—numerous centres in Poland order free performance of prenatal diagnostics and take the risk of falsifying the medical record in such a way so as to guarantee that the state refunds the benefit.^3^

Our study also revealed that a significant percentage of geneticists (slightly above 25 per cent) still associate performing invasive prenatal testing with the possibility of deciding on pregnancy termination. These persons believed that for women who want to know the fetal health status and declare that they would not terminate the pregnancy under any circumstances, invasive prenatal testing should not be carried out. It is difficult to understand the underlying motivation of the participants, but the following reasons might be at play: 1) care for the good of the fetus and unwillingness to expose it to the unnecessary risk associated with the prenatal examination, 2) conviction that the main objective and justification for performing such testing is the detection and elimination of the highest possible number of fetuses with malformations, and 3) conviction that the state budget should not cover the cost of an examination the sole aim of which is to satisfy the curiosity of a pregnant woman. However, further research is required for establishing the reason behind rejecting invasive prenatal testing for women who declare their intent to maintain the pregnancy.

The results of earlier studies demonstrated that assigning the importance of nondirectiveness and its translation into practice can be affected by sex, whereas our results did not reveal such a relationship. The surveyed men frequently assigned importance to directiveness equally as the women, and they often equally recognized that it is not absolute. All the surveyed men accepted invasive prenatal testing in the situation when a woman declares maintaining the pregnancy irrespective of the test outcome. Thus, these results are not coherent with those previously cited indicating differences between the sexes (Curlin et al. 2007; Wertz 1994; Wertz and Fletcher 1989).

The study further points to the potential role of professional experience and the nature of the workplace in the acceptability of directiveness. People with lower professional experience and those working at commercial institutions accepted directiveness to a greater degree. However, these results should be analysed further to understand if over time the nondirectiveness models provided during education have changed and if the institution’s assumptions as well as patients’ expectations toward their physician employed at private institutions differ from those of the national institutions and how they affect the view on professional conduct.

The level of professional experience also seemed to be important in indicating which malformation is severe enough to obtain the certificate authorizing pregnancy termination. This can be explained by factors uncontrolled during the study (e.g., general attitude toward pregnancy termination). Pediatrics specialization was also linked to the less common assessment of Down syndrome as a sufficient disorder for issuing the statement. This may be explained by the fact that the basic patient for a pediatrician is a child. Assessment of disorder severity may, in this case, be predominantly driven by the assumed possible psychological wealth of the child and the relatively mild physical impairment. The burden of parents caring for children with special needs may be downplayed or given less weight.

A significant finding of the study was that abstract declarations of the geneticists concerning the appropriate regimen do not always translate into their choices in specific clinical situations. This phenomenon was observed for the above-discussed discrepancy between declarations of attachment to nondirectiveness value and the general acceptance for not using the principle in practice, as well as for the declaration on the inappropriateness of performing invasive prenatal testing in those pregnant women who declared that they would not terminate the pregnancy. In the latter case, close to ninety per cent of the surveyed geneticists who indicated that they would not issue a referral for the examination would act differently than declared in a situation similar to clinical practice. It is difficult to determine the reasons for this discrepancy, but the following two hypotheses seem plausible: 1) discrepancy between what one should declare (as a commitment to professional standard) and one’s own actions; and 2) lack of trust in the permanence of the patient’s decision on maintaining the pregnancy after receiving information on fetal malformation.

The results of the study showed that the surveyed geneticists were greatly reluctant to take personal responsibility for issuing a certificate authorizing a woman to terminate the pregnancy. The majority wished that such decisions were made collectively or that there was a list of malformations published by the legislature authorizing pregnancy termination. This may stem from the reluctance of Polish physicians to be directly and personally associated with pregnancy termination procedures. It is worth noting that the wish to have a definitive list of conditions when termination is legal is neither realistic nor ethical. Many of the genetic conditions detected in a fetus have vast range of manifestations. The very diagnosis is an imperfect predictor of severity of symptoms, level of physical and mental disability, and quality of life of the child to be born.

Our study documents that Polish physicians have become very cautious due to the highly conservative climate that has prevailed in the country for many years and numerous attempts that were made to tighten the abortion law. They prefer to stand aside and not get involved in actions aiming at pregnancy termination for the fear of facing problems.^4^ The obtained results showed that Polish geneticists do not believe that the possibility of terminating pregnancy if a fetal malformation is identified is a positive right of a woman. Only a minor percentage was inclined to involve in any way the pregnant woman in the process of making decision on the further fate of pregnancy.

Our study further revealed that geneticists have a highly significant impact on the actual possibility of a pregnant woman deciding on terminating the pregnancy if a fetal malformation is determined. Due to the recent, intensifying anti-abortion campaign in Poland and the regular attempts to tighten the law in this area, Polish geneticists have also taken a harsh course and are trying to limit access to abortion in cases they believe to be considered “problematic” in the current political climate.

In addition, our study indicated the existence of a large group of geneticists who believe that even severe fetal malformations linked to profound physical and mental disability do not justify issuing a referral authorizing a woman to terminate the pregnancy. The greatest surprise was the fact that several surveyed individuals did not see the basis for issuing the certificate authorizing pregnancy termination even in the case of Edwards’ syndrome which is characterized by lethal malformations (incompatible with life).

Thanks to the use of a methodology that combined closed-ended with open-ended questions and confronting the rules of conduct declared by the surveyed with their actual actions in situations similar to clinical cases, we succeeded in showing that the declarative attachment to the idea of nondirectiveness is not always accompanied by the use of this principle in practice. The study also provided evidence that some geneticists, through their actions, limit the range of choices available to patients (which was particularly apparent in the case of reproductive choices) and still believe that they act in line with the professional standard. Strong medical paternalism manifests itself particularly in invasive prenatal examinations where the geneticists play the role of gatekeepers. A vast majority of the surveyed geneticists thought that the decision on issuing a referral for an invasive prenatal examination should depend on the existence of objective reasons for its performance (which was understood as the likelihood of fetal malformation). The willingness of a pregnant woman to identify the fetal health status in the absence of other medical indications was assumed by a considerable majority of geneticists as an insufficient reason for issuing a referral for invasive prenatal testing. Perhaps, partial responsibility for this state of affairs is borne by the unfortunate wording included in both professional standards and legal provisions, according to which a physician orders the performance of prenatal examination. This may suggest to many physicians that the decision concerning the performance of invasive prenatal testing belongs to them and should be based solely on medical criteria, while the will of a pregnant woman to know the health status of the fetus is not important in this situation.^5^

## Limitations

Study results cannot be generalized to all Polish geneticists, despite the study involving a large percentage of this targeted group. It is possible that persons who did not participate are characterized by a different distribution of attitudes than that observed in the study. Although clinical geneticists in Poland form a rather small group and a tight community, it cannot also be excluded that some of the persons did not participate in the study for the fear of being identified from the demographic information provided in the survey (age, sex, experience, additional specialty). Authors in numerous contacts with the geneticists’ community prior to the survey emphasized its anonymous nature. However, the sole fact that such concerns were expressed may indicate the high distrust of the community toward any studies that reveal their views and attitudes. Particular distrust may characterize persons with extreme opinions or with those not generally accepted in the community. Despite those reservations about representativeness, the obtained results show variable attitudes, ranging from strongly liberal attitudes toward women’s rights to decide on the fate of pregnancy to those that are strongly conservative and paternalistic.

Another limitation of the study is its incapability to strictly predict how the attitudes and choices presented by the counsellors would translate into their practical action. There is no way of proving that people actually do what they say they would do. Neither can we assume that the responses would be the same if geneticists and genetic counsellors were presented with cases concerning testing for genetic conditions in adults.

## Footnotes

1 The Ordinance of the Minister of Health of 6 November 2013 on health programmes financed from public funds Dz.U. [Journal of Laws] from 2013, item 1505, as amended.

2 Article 4a.1 of the 1993 Act of January 7, 1993, on Family Planning, Protection of the Human Fetus and Conditions Permitting Pregnancy Termination, Dz.U. [Journal of Laws] from 1993, no. 17, item 78. After the submission of the article the law in Poland was furthered tightened. The Constitutional Court ruled that allowing abortions due to fetal defects is unconstitutional, banning one of the last remaining legal grounds for the procedure in Poland (The Judgment from 22 of October 2020 (K1/20) - https://ipo.trybunal.gov.pl/ipo/Sprawa?sprawa=22412&dokument=20359&cid=1)

3 This thesis has been based on the observations made in genetic clinics and the information provided to us by their employees.

4 By problems, we mean being harassed by pro-life activists, being publicly called “a mass murderer,” being subjected to long and unpleasant institutional and/or legal investigations, etc. For more on the climate around prenatal examination and termination of pregnancy in Poland, see Różyńska and Chańska (2013).

5 This conviction is against the Polish law that provides a pregnant woman with subjective right to reliable information on the health status and pregnancy hazards and the right to referral for genetic prenatal testing. This opinion, expressed by the Supreme Court on several occasions (e.g., in the judgment for October 13, 2005 signature IV CK 161/05), is still not shared by a considerable portion of the medical community.
